# Proteomic-based biomarker discovery reveals panels of diagnostic biomarkers for early identification of heart failure subtypes

**DOI:** 10.1186/s12967-025-06563-7

**Published:** 2025-05-15

**Authors:** Narainrit Karuna, Claire Tonry, Mark Ledwidge, Nadezhda Glezeva, Joe Gallagher, Ken McDonald, Chris J. Watson

**Affiliations:** 1 Wellcome-Wolfson Institute for Experimental Medicine, Queen’s University Belfast, Belfast, UK; 2 Faculty of Pharmacy, Chiang Mai University, Chiang Mai, Thailand; 3 UCD Conway Institute and Research and Innovation Programme for Chronic Disease, School of Medicine, University College Dublin, Dublin, Ireland; 4 STOP-HF Unit, Department of Cardiology, St. Vincent’s University Healthcare Group, Dublin, Ireland

## Abstract

**Background:**

Limited access to echocardiography can delay the diagnosis of suspected heart failure (HF), which in turn postpones the initiation of optimal guideline-directed medical therapy. Although natriuretic peptides like B-type natriuretic peptide (BNP) are valuable biomarkers for diagnosing and managing HF, the utility of combining BNP with other blood-based biomarkers to predict subtypes of new-onset HF remains underexplored.

**Objectives:**

This study sought to investigate and evaluate the diagnostic significance of adding blood-based biomarkers to BNP for identifying heart failure with preserved ejection fraction (HFpEF) or reduced ejection fraction (HFrEF), with the goal of enhancing diagnostic assays beyond BNP measurements.

**Methods:**

We identified candidate blood protein biomarkers using untargeted proteomics workflows from a cohort of individuals recruited to the STOP-HF trial who were at risk of HF and subsequently developed either HFpEF or HFrEF over time (“HF progressors”; n = 40). Candidate biomarkers were verified in an independent cohort (n = 52) from a community-based rapid access HF diagnostic clinic. The biological processes associated with these proteins were assessed, and the diagnostic values of biomarker panels were evaluated using a machine learning approach.

**Results:**

Within HF progressors, we identified 3 proteins associated with HFpEF development: vascular cell adhesion protein 1 (VCAM1), insulin-like growth factor 2 (IGF2), and inter-alpha-trypsin inhibitor heavy chain 3 (ITIH3). Additionally, 4 proteins were linked to HFrEF development: C-reactive protein (CRP), interleukin-6 receptor subunit beta (IL6RB), phosphatidylinositol-glycan-specific phospholipase D (PHLD), and noelin (NOE1). These findings were verified in an independent cohort to distinguish HF subtypes from controls. Moreover, a random forest algorithm demonstrated that combining these candidate biomarkers with BNP measurement significantly improved the prediction of HF subtypes.

**Conclusions:**

We identified candidate proteins linked to HFpEF and HFrEF in a longitudinal HF progressor cohort and validated them in a community-based cohort. Adding these proteins to BNP led to a significant improvement in HF subtype prediction. Study results have clinical implications for blood-based screening of HF subtypes using panels of biomarkers, particularly in resource-limited settings.

**Supplementary Information:**

The online version contains supplementary material available at 10.1186/s12967-025-06563-7.

## Introduction

Development of heart failure (HF) in community patients does not occur suddenly but rather in a continuum from pre-HF to symptomatic HF [1, 2]. Despite recent progress in HF management, both for HF with reduced ejection fraction (HFrEF) and HF with preserved ejection fraction (HFpEF), the rates of mortality and re-hospitalisation continue to increase at an alarming rate [3]. Risk stratification and early diagnosis of non-acute HF are important in ensuring that optimal guideline-directed medical therapy (GDMT) is initiated to prevent or slow down further progression [4, 5]. Patients suspected of HF development are generally assessed by clinical assessment with history and physical examination; however, isolated symptoms and signs may poorly correlate with cardiac function and performance [6–8]. Although non-invasive imaging techniques, such as echocardiography, can provide substantial evidence for identifying or excluding HF, limited access to echocardiography poses significant challenges for diagnosing new-onset HF and delaying treatments, especially in primary care settings [9, 10].

Natriuretic peptides, including plasma B-type natriuretic peptide (BNP) and the inactive N-terminal counterpart (NT-proBNP), are useful biomarkers to support diagnosis of HF and are incorporated into HF clinical practice guidelines [9–12]. Furthermore, the STOP-HF (St Vincent’s Screening to Prevent Heart Failure) study, a large single-centre trial of patients at risk of HF, underlined the utility of BNP-based screening and collaborative care in reducing left ventricular (LV) dysfunction and newly diagnosed HF [13]. Development of HF involves several mechanisms and interplay amongst pathogenic drivers such as neurohormonal activation, inflammation, LV remodelling, myocyte injury and alteration of the extracellular matrix [14, 15]. Beyond the natriuretic peptides, other circulating protein biomarkers may provide valuable insights into the initial physiological alterations and biological signature that contribute to the subsequent HF development, with the potential to enhance understanding of the uniqueness of pathogenesis of HFrEF and HFpEF. Consequently, the combination of BNP measurements with other blood-based biomarkers to predict subtypes of new-onset HF is unknown. We set out to identify and determine the diagnostic value of multiple blood-based biomarkers derived from patients who progress from pre-HF to either HFpEF or HFrEF, when combined with BNP measurements to establish novel panels of diagnostic and prognostic HF biomarkers.

## Methods

Detailed methods are presented in the supplementary material online.

### Study population (HF-progressors)

The STOP-HF trial [13] was a successful screening programme using natriuretic peptide measurement and collaborative care in an at-risk population to reduce newly diagnosed HF and the prevalence of significant LV systolic and/or diastolic dysfunction. For the purpose of analyses to reveal biomarkers predicting HF phenotypes in the community setting, we retrospectively investigated patients who did not have HF at baseline but subsequently developed new-onset HF over time, compared to a group who did not develop HF over a similar time period. Patients (n = 40) from the STOP-HF trial cohort were used in this study; n = 14 new-onset HFrEF (baseline and follow-up); n = 14 new-onset HFpEF (baseline and follow-up); and n = 12 No-HF (baseline and follow-up). The inclusion and exclusion of participants have been described in more detail previously [13].

### Independent verification cohort (community-based)

We recruited 52 patients with signs of breathlessness and suspected HF from primary care who attended a rapid access HF diagnostic clinic, which was established to support early and accurate diagnosis of HF [16]. These patients were assessed by the HF clinical team and were classified into HFrEF (n = 12), HFpEF (n = 22), and no HF (‘Control’; n = 18) based on European Society of Cardiology diagnostic criteria. In brief, the diagnosis of HF was based on typical symptoms (with or without signs of fluid overload), elevated BNP levels, and confirmed by echocardiography. The full details of patient flow in the rapid access HF diagnostic clinic and diagnosis criteria are previously reported [16].

### Sample collection and processing

Among patients diagnosed with either HFrEF or HFpEF and No-HF within the STOP-HF trial cohort, previously biobanked EDTA plasma samples were collected at baseline and the follow-up visit before the HF diagnosis event. Samples for independent verification were collected from community referred patients to the rapid access HF diagnostic clinic in St Vincent’s University Hospital, Dublin. For each patient, blood samples were collected in EDTA tubes and then immediately centrifuged at 2500*g* for 10 min at 4 °C to isolate plasma. Afterwards, the plasma was divided into aliquots and stored at − 80 °C until further processing.

### Plasma depletion and processing

Aliquots of 30 µl of human plasma were depleted of the top 14 most abundant plasma proteins using High-Select™ Top14 Abundant Protein Depletion Resin (A36372, Thermo Scientific™). Plasma samples were denatured and digested by 8 M urea/10 mM Tris–HCl (pH 8) and subsequently quantified by a bicinchoninic acid assay (BCA, Pierce–Thermo Scientific™) for protein concentration. After that, plasma protein samples were dissolved in 50 mM ammonium bicarbonate (pH 7.8)/100 mM dithiothreitol (DTT) and incubated at 27 °C for 1 h. Iodoacetamide (IAA) at 140 mM was added to plasma protein samples and incubated for 30 min in the dark at room temperature. Plasma protein samples were quenched by adding DTT and diluting the reaction with 50 mM ammonium bicarbonate to reduce the final urea concentration to lower than 2 M. Plasma protein samples underwent trypsin (Sequencing Grade Modified Trypsin, V5111, Promega UK) digestion (enzyme-to-substrate ratio of 1:50 at 37 °C for overnight), and the reaction was terminated by adding trifluoroacetic acid (TFA) to a final concentration of 1%. The peptides were then extracted and dried. Digested samples were cleaned on C18 stage tips and dried in a vacuum centrifuge. Samples were reconstituted with 25 µl of Solvent A (0.1% formic acid). Sample concentrations were normalised based on the absorbance at 205 nm measured with a nanodrop (Thermo Scientific). Pooled samples were prepared at the end/beginning of the sample digest procedure to monitor technical variability.

### LC–MS data acquisition

The samples were analysed using an Evosep One chromatography system (30 SPD runs) coupled to a tims-TOF Pro mass spectrometer (Bruker Daltonics). Samples (~ 1 µg) in Solvent A were directly injected into the C18 PepSep column (15 cm × 100 μm ID, C18, 3 μm). Mobile phases were 0.1% (v/v) formic acid in water (phase A) and 0.1% (v/v) formic acid in acetonitrile (phase B). The peptides were separated by an increasing gradient of mobile phase B for 44 min using a flow rate of 0.5 µl/min. The mass spectrometer was operated in either data dependent acquisition (dda)-Parallel Accumulation-Serial Fragmentation (PASEF) mode for pooled samples or data independent acquisition (dia)-PASEF mode for individual samples.

### Proteomic analysis

FragPipe computational platform (v20.0) with MSFragger (v3.8) and Philosopher (v5.0) were used to build spectral libraries using dda-acquired data [17–19]. Protein sequence databases *H. sapiens* (UP000005640) from UniProt were used (https://www.uniprot.org/proteomes/UP000005640). Then, DIA-NN (v1.8.1) pipeline [20] was applied using dia-acquired data to search proteins against the spectral library with a false discovery rate (FDR) of 1%. The mass accuracy tolerances were set to 15 ppm for both MS1 and MS2 spectra. Quantification mode was set to “Robust LC (high precision)”. All other settings were left default, following previously published recommendations [21], and similarly to the previous dia-PASEF workflow [22].

Log 2 transforming was applied to the resulting data, and any proteins observed in less than 50% of the samples were not included in subsequent analyses. The dataset was normalised by LOESS from the limma package [23]. The rest of the missing values were imputed using random draws from a manually defined left-shifted Gaussian distribution (shift = 1.8, scale = 0.3).

Over-representation analysis (ORA) was performed to identify the biological process associated with the significantly differentially expressed proteins in subtypes of HF from baseline (P < 0.05) using clusterProfiler [24]. The parameters were set as following: minGSSize = 2, maxGSSize = 500, pvalueCutoff = 0.05, ont = “BP”. The results were plotted using SRplot platform [25].

### Biomarker models

Machine learning analyses were performed using the tidymodels package (v1.2; https://CRAN.R-project.org/package=tidymodels). In order to minimise the chance of overfitting, estimate prediction performance, and tune model hyperparameters, fivefold cross-validation was used. Naive Bayes (NB) [26], multivariate adaptive regression splines (MARS) [27], and random forest (RF) [28] were implemented to find the best classifier fitted to the dataset. Data from the independent community-based cohort was used as input data, and the dataset was split into 60% training dataset and 40% testing dataset. Model parameters with or without hyperparameter tuning were estimated on the training data, and the best-trained models based on the area under the receiver operating characteristic curve (AUC) during cross-validation were tested on the testing dataset. Receiver Operating Characteristic (ROC)/AUC analysis was utilised to assess the diagnostic performance and accuracy of the prediction models.

### Statistical analysis

GraphPad (v10) and R programme 4.4.0 with relevant packages were used for data analysis. Unpaired t-test or Mann–Whitney test was used for independent continuous variables, and Wilcoxon matched-pairs signed rank test or Paired t-test was used for dependent continuous variables, as appropriate. χ^2^ or Fisher exact test was used for categorical variables as appropriate. All statistical tests are 2-tailed, and statistical significance was defined as P < 0.05.

## Results

### Characteristics of HF-progressors cohort

**Fig. 1:**
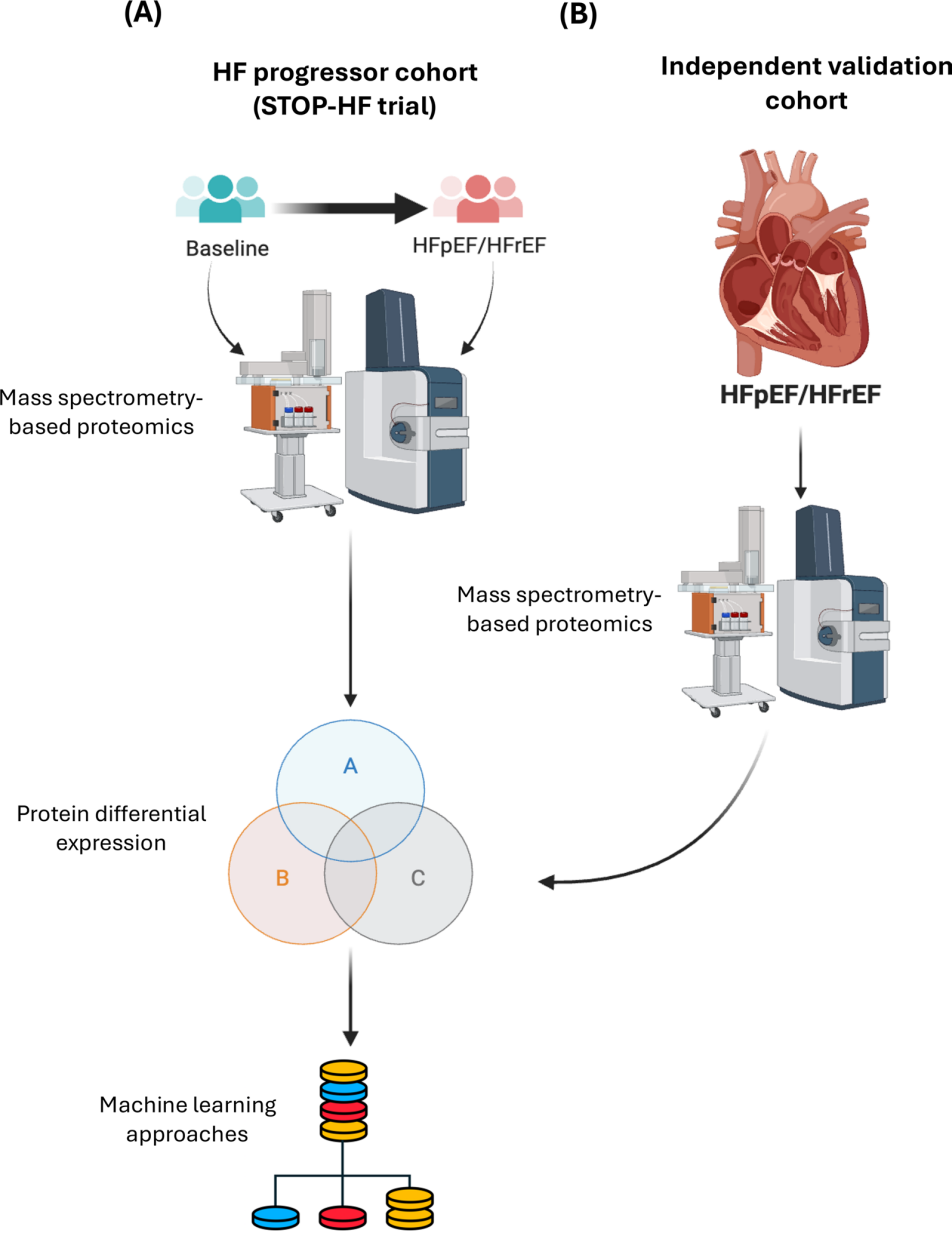
Study design of proteomics-based biomarker discovery. **A** HF progressor cohort (N = 40). Blood samples are collected at baseline and diagnosis of no-HF (N = 12) or HFpEF (N = 14) or HFrEF (N = 14). **B** Independent community-based cohort (N = 52). Blood samples of patients diagnosed with HFpEF (N = 22), HFrEF (N = 12), and control (N = 18) are collected. HFpEF = heart failure with preserved ejection fraction; HFrEF = heart failure with reduced ejection fraction

At baseline, a total of 40 participants from the STOP-HF trial (HF-progressors) (mean age ± SD = 74.0 ± 6.24 years, male = 50%; Table 1) were selected for blood-based biomarkers discovery for identifying subtypes of HF. Selected patients did not have HF at baseline but subsequently developed new-onset HFpEF (n = 14), new-onset HFrEF (n = 14) over time, and No-HF (n = 12). This HF progressors cohort was studied using mass-spectrometry-based proteomics to reveal signature proteome changes over time from pre-HF to symptomatic HF (Fig. 1A). Elevated BNP was evident in HFpEF (P = 0.0107) and HFrEF (P = 0.0006), compared to baseline, whilst there was no difference in the No-HF group (P = 0.7120; Table 1). Patients with HFrEF (mean EF ± SD = 38.1 ± 6.96%) showed significantly decreased EF from baseline (mean EF ± SD = 47.1 ± 9.88%; Table 1).

**Table 1:** Characteristics of heart failure (HF)-progressors from STOP-HF cohort

	No-HF		P-value (no-HF) (baseline vs Dx)	HFpEF		P-value (HFpEF) (baseline vs Dx)	HFrEF		P-value (HFrEF) (baseline vs Dx)	Overall	
	Baseline	Dx		Baseline	Dx		Baseline	Dx		Baseline	Dx
	(n=12)	(n=12)		(n=14)	(n=14)		(n=14)	(n=14)		(n=40)	(n=40)
Gender											
Female	6	6		7	7		7	7		20	20
(%)	(50.0%)	(50.0%)	>0.9999	(50.0%)	(50.0%)	>0.9999	(50.0%)	(50.0%)	>0.9999	(50.0%)	(50.0%)
Male	6	6		7	7		7	7		20	20
(%)	(50.0%)	(50.0%)		(50.0%)	(50.0%)		(50.0%)	(50.0%)		(50.0%)	(50.0%)
Age (years)											
Mean	74.4	76.1		75.0	76.9		72.7	74.5		74.0	75.8
(SD)	(4.61)	(4.31)	**0.0005**	(6.46)	(6.44)	**0.0001**	(7.35)	(7.36)	**<0.0001**	(6.24)	(6.18)
Median	72.8	74.4		76.1	78.1		72.7	74.3		74.2	75.8
[IQR]	[4.52]	[3.20]		[3.30]	[3.40]		[8.16]	[8.47]		[6.06]	[5.87]
EF (%)											
Mean	68.3	68.9		69.1	66.6		47.1	38.1		61.2	57.3
(SD)	(6.61)	(5.33)	0.7251	(7.39)	(6.01)	0.2129	(9.88)	(6.98)	**0.0007**	(13.1)	(15.5)
Median	68.0	70.0		69.5	67.0		50.0	39.0		62.5	62.0
[IQR]	[6.25]	[5.50]		[14.3]	[9.25]		[11.9]	[9.25]		[20.3]	[25.8]
BNP (pg/ml)											
Mean	37.6	40.6		111	210		130	382		95.4	220
(SD)	(24.2)	(14.7)	0.712	(75.2)	(175)	**0.0107**	(83.8)	(379)	**0.0006**	(76.9)	(278)
Median	29.1	42.5		84.4	154		136	240		70.7	128
[IQR]	[27.6]	[18.6]		[88.4]	[107]		[104]	[285]		[105]	[188]

Chi-squared test is used for categorical variables, and paired t test or Wilcoxon matched-pairs signed rank test is used for numerical variables

*Dx* diagnosis, *EF* ejection fraction, *BNP* b-type natriuretic peptide, *No-HF* no heart failure, *HFpEF* heart failure with preserved ejection fraction, *HFrEF* heart failure with reduced ejection fraction, *SD* standard deviation, *IQR* interquartile range

### Characteristics of independent verification cohort (community-based)

There were 52 participants in this cohort (Table 2; Fig. 1B), including HFpEF (n = 22), HFrEF (n = 12), and controls (n = 18). Patients in the cohort were on average 77.3 ± 9.92 (mean ± SD) years and 38.5% male. HFrEF patients had considerably lower EF compared to controls [mean EF ± SD = (37.4 ± 10.5% vs 65.4 ± 5.82%; P < 0.0001)], whereas no differences between HFpEF and controls were evident (P = 0.4362; Table 2). Blood BNP levels were increased in HFpEF (224 ± 114 vs 55.1 ± 71.1 pg/ml; P < 0.0001) and HFrEF (223 ± 155 vs 55.1 ± 71.1 pg/ml; P = 0.0007), compared to controls, respectively. Of the 52 participants, 69.2% had hypertension, 46.2% had dyslipidaemia, and 34.6% had arrhythmia (Table 2). Participants were using antihypertensive drugs [n = 47 (90.4%)], beta-blockers [n = 28 (53.8%]), loop diuretics [n = 25 (48.1%)], and aspirin [n = 23 (44.12%)] (Table 2). Other details are presented in Table 2.

**Table 2:** Characteristics of independent verification cohort (community-based)

	Control	HFpEF	HFrEF	P-value (HFpEF vs control)	P-value (HFrEF vs control)	Overall
	(n=18)	(n=22)	(n=12)			(n=52)
Gender						
Female (%)	12 (66.7%)	15 (68.2%)	5 (41.7%)	0.9189	0.1758	32 (61.5%)
Male (%)	6 (33.3%)	7 (31.8%)	7 (58.3%)			20 (38.5%)
Age (years)						
Mean (SD)	75.4 (8.61)	81.5 (7.33)	72.6 (13.2)	**0.0221**	0.4775	77.3 (9.92)
Median [IQR]	75.5 [8.00]	82.0 [8.50]	73.0 [22.0]			79.0 [11.5]
Weight (kg)						
Mean (SD)	83.0 (15.2)	81.3 (19.9)	84.5 (22.1)	0.7746	0.8232	82.6 (18.6)
Median [IQR]	82.9 [23.1]	83.3 [32.5]	81.0 [41.6]			82.6 [30.2]
HR (BPM)						
Mean (SD)	70.9 (16.8)	75.4 (16.2)	75.2 (9.45)	0.2947	0.211	73.8 (15.0)
Median [IQR]	65.5 [20.8]	74.0 [19.3]	77.5 [12.0]			72.0 [18.5]
SBP (mmHg)						
Mean (SD)	147 (21.0)	153 (19.6)	144 (26.1)	0.4198	0.4208	149 (21.5)
Median [IQR]	144 [29.8]	154 [25.8]	142 [27.3]			150 [27.3]
DBP (mmHg)						
Mean (SD)	71.1 (14.8)	73.9 (15.1)	73.7 (15.7)	0.5578	0.6476	72.8 (14.9)
Median [IQR]	72.5 [15.8]	72.0 [21.0]	72.5 [17.8]			72.0 [19.3]
EF (%)						
Mean (SD)	65.4 (5.82)	63.8 (6.40)	37.4 (10.5)	0.4362	**<0.0001**	57.7 (13.9)
Median [IQR]	67.0 [8.00]	64.5 [6.25]	39.5 [16.3]			62.0 [15.8]
Missing (%)	2 (11.1%)	2 (9.1%)	0 (0%)			4 (7.7%)
BNP (pg/ml)						
Mean (SD)	55.1 (71.1)	224 (114)	223 (155)	**<0.0001**	**0.0007**	165 (137)
Median [IQR]	25.5 [42.7]	201 [161]	231 [274]			135 [205]
Na (mEq/l)						
Mean (SD)	140 (2.85)	139 (4.09)	139 (2.73)	0.7503	0.6781	139 (3.37)
Median [IQR]	140 [2.00]	140 [5.75]	140 [1.25]			140 [3.50]
K (mEq/l)						
Mean (SD)	4.29 (0.310)	4.15 (0.434)	4.33 (0.452)	0.258	0.8271	4.24 (0.399)
Median [IQR]	4.35 [0.450]	4.15 [0.525]	4.20 [0.375]			4.25 [0.425]
CL (mEq/l)						
Mean (SD)	105 (2.62)	102 (5.62)	104 (4.37)	**0.0369**	0.3447	104 (4.62)
Median [IQR]	106 [2.50]	103 [6.75]	105 [4.25]			105 [5.25]
Urea (mmol/l)						
Mean (SD)	7.26 (2.73)	8.32 (3.64)	9.14 (2.41)	0.3147	**0.0375**	8.14 (3.12)
Median [IQR]	6.65 [2.30]	7.40 [3.08]	9.40 [3.33]cl			7.40 [3.85]

Chi-squared test or Fisher’s exact test is used for categorical variables, and unpaired t test or Mann Whitney test is used for numerical variables

*HFpEF* heart failure with preserved ejection fraction, *HFrEF* heart failure with reduced ejection fraction, *HR* heart rate, *SBP* systolic blood pressure, *DBP* diastolic blood pressure, *EF* ejection fraction, *BNP* b-type natriuretic peptide, *Na* sodium, *K* potassium, *CL* chloride. *E/A ratio* peak early to late diastolic filling velocity, *SD* standard deviation, *IQR* interquartile range

### Biomarkers association with new-onset HFpEF

**Fig. 2:**
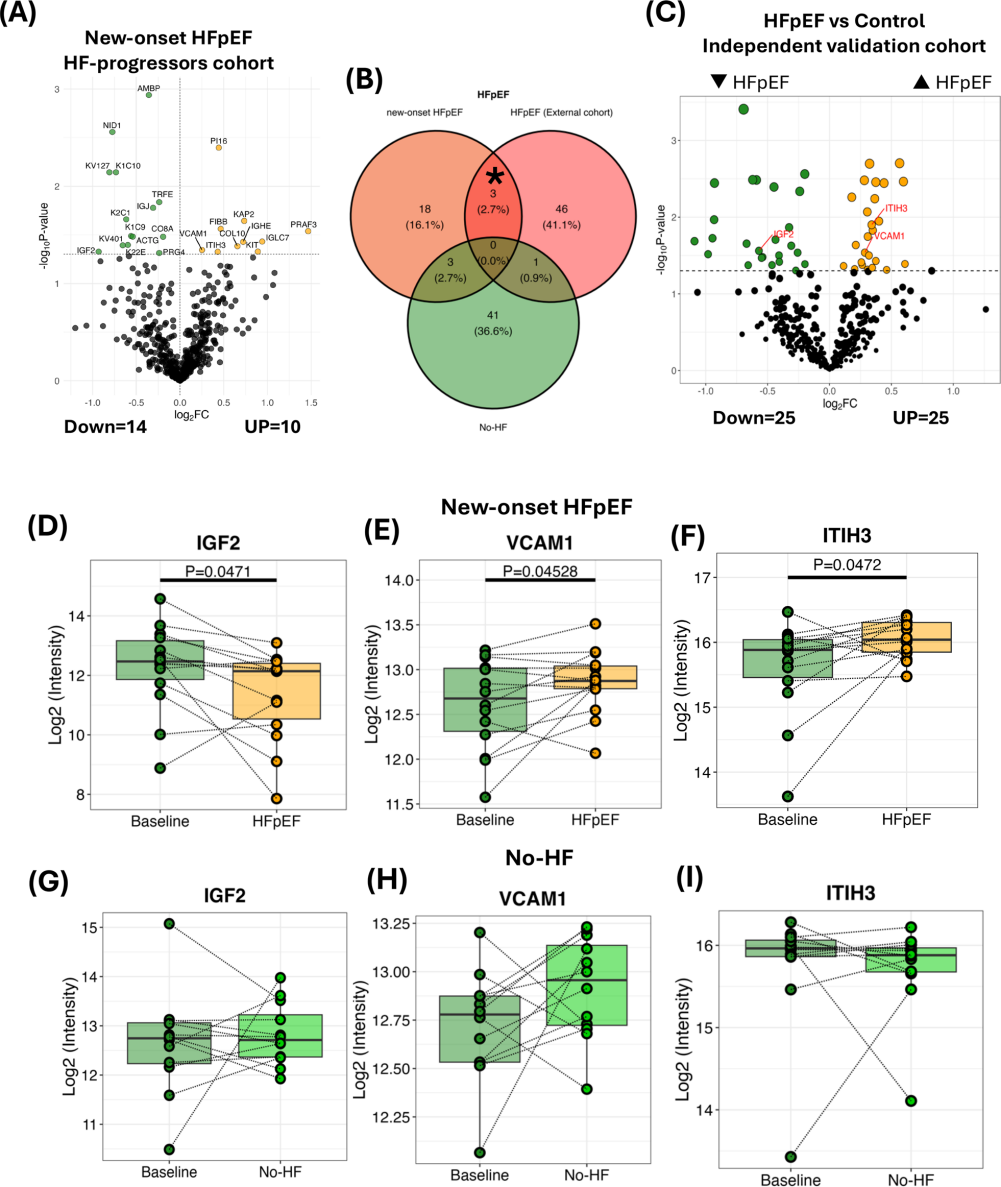
Biomarkers associated with HFpEF development. **A** Volcano plot of new-onset HFpEF, compared to baseline (yellow = up-regulated proteins; green = down-regulated proteins). **B** Venn diagram of differential protein expression (P < 0.05) for new-onset HFpEF, No-HF, and independent community HFpEF cohort. **C** Volcano plot of HFpEF vs control patients in independent community HFpEF cohort. **D**–**F** Co-expression proteins in new-onset HFpEF patients from (**B**) between new-onset HFpEF and independent community HFpEF cohort. **G**–**I** Co-expression proteins in No-HF patients from (**B**) between new-onset HFpEF and independent community HFpEF cohort. HFpEF = heart failure with preserved ejection fraction; No-HF = no heart failure; IGF2 = insulin like growth factor 2; VCAM1 = vascular cell adhesion protein 1; ITIH3 = inter-alpha-trypsin inhibitor heavy chain 3. *Overlap protein expression in same direction between new-onset HFpEF and independent community HFpEF cohort

There were 570 proteins identified in the HF-progressors dataset, and 489 proteins passed the pre-processing stage. We found 10 up-regulated proteins and 14 down-regulated proteins associated with HFpEF development (Fig. 2A). To further evaluate these plasma proteomes, we carried out single time point proteomics analysis in the independent community-based cohort comparing patients newly diagnosed with HFpEF and controls (Fig. 2C). The results demonstrated 3 plasma proteins, including vascular cell adhesion protein 1 (VCAM1), insulin like growth factor 2 (IGF2), and inter-alpha-trypsin inhibitor heavy chain 3 (ITIH3) that were overlapping proteins between the HF-progressors and independent community-based cohort (HFpEF vs control) (Fig. 2B, C). In new-onset HFpEF, plasma IGF2 was significantly decreased at the time of diagnosis (P = 0.0471; Fig. 2D), while plasma VCAM1 and ITIH3 were increased in new-onset HFpEF patients, compared to the baseline (Fig. 2E, F). These proteins did not significantly change in No-HF group (all P > 0.05; Fig. 2E,G. F).

Furthermore, ORA was performed to gain insight into the biological processes of these proteins related to HFpEF development (Supplement Fig. 1). IGF2 was involved in tissue development, organelle organization, immune system process, cell differentiation and cellular processes (organization or biogenesis) (Supplement Fig. 1A). Organonitrogen compound metabolic process was shared for ITIH3 and VCAM1, while VCAM1 was enriched in several pathways associated with response to stimulus and intracellular signalling processes (Supplement Fig. 1B).

**Fig. 3:**
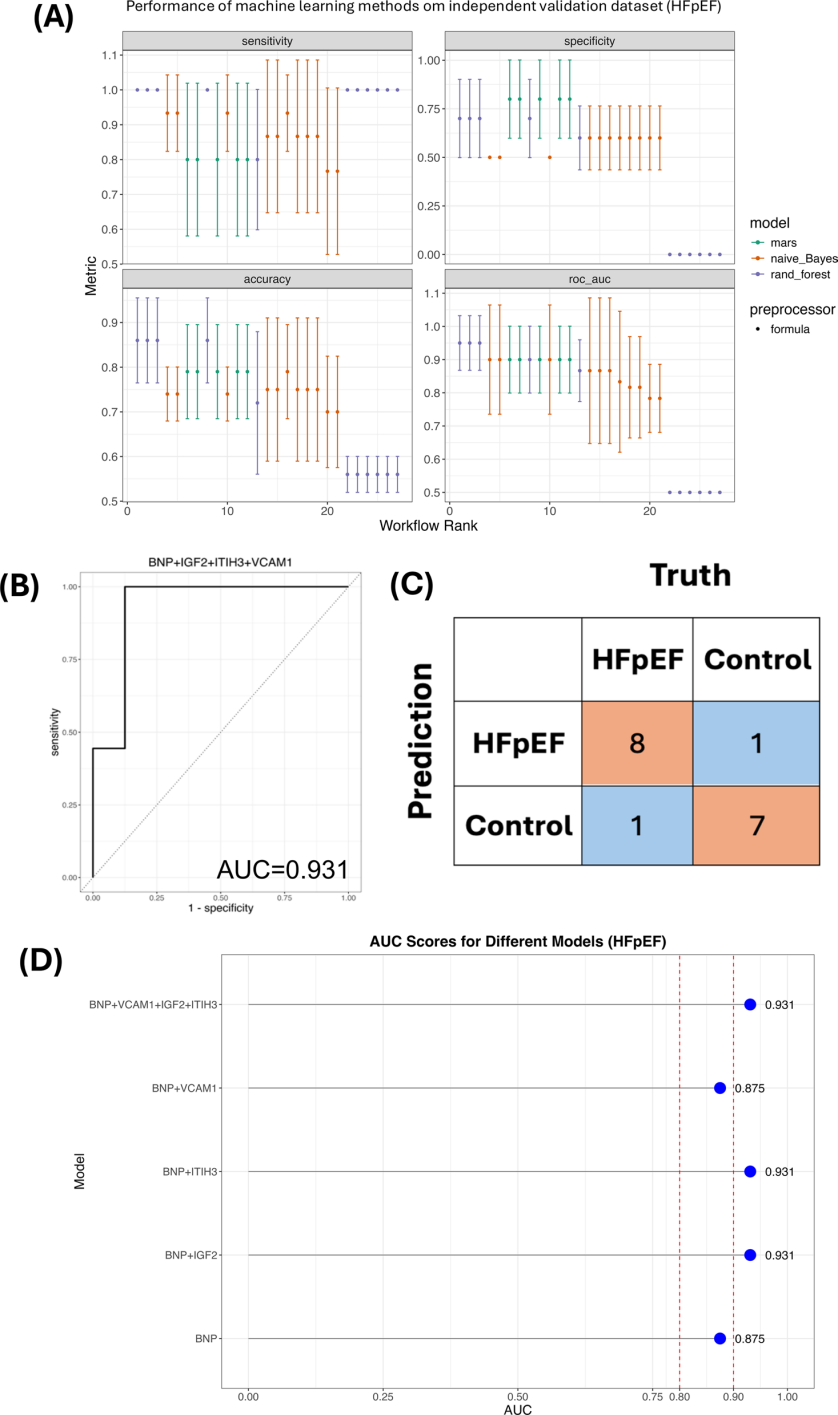
Predictive accuracy of plasma proteins related to HFpEF, alone or in combination with BNP measurement in independent community HFpEF cohort. **A** Models performance based on naive Bayes (NB), multivariate adaptive regression splines (MARS), and random forest in training dataset. **B** Receiver operating curves show the performance of combining all candidate protein biomarkers with BNP measurement for predicting all incident HFpEF in testing dataset. **C** Confusion matrix of (**B**). **D** AUC of different combination or BNP alone in testing dataset. HFpEF = heart failure with preserved ejection fraction; mars = multivariate adaptive regression splines; rand_forest = random forest; AUC = area under the curve; IGF2 = insulin like growth factor 2; VCAM1 = vascular cell adhesion protein 1; ITIH3 = inter-alpha-trypsin inhibitor heavy chain 3

Machine learning algorithms were applied in the independent community-based cohort to study the performance of IGF2, VCAM1, and ITIH3, when combined with BNP, for classifying HFpEF development from controls. Performance metrics for all algorithms ranked by AUC and RF showed the best results concerning AUC, followed by NB and MARS (Fig. 3A). Our RF algorithm was applied to the dataset, and the AUC of BNP + VCAM1 + IGF2 + ITIH3 was 0.931 (Fig. 3B). The confusion matrix of the RF model accurately classified all of the HFpEF patients, with one misclassification observed for the negative class (healthy controls) and the positive class (HFpEF) (Fig. 3C). Furthermore, the performance of screening HFpEF patients with BNP alone was examined using the RF algorithm, and the result indicated that BNP fairly performed to classify HFpEF patients (AUC = 0.875) (Fig. 3D). The AUC of BNP + ITIH3 and BNP + IGF2 were 0.931; however, BNP + VCAM1 did not add capabilities when compared with BNP alone (AUC = 0.875) in our analysis (Fig. 3D). Taken together, these results highlight the utility of multiplexed biomarker panels beyond BNP alone for the improvement of HFpEF diagnosis using blood-based markers.

### Biomarker association with new-onset HFrEF

**Fig. 4:**
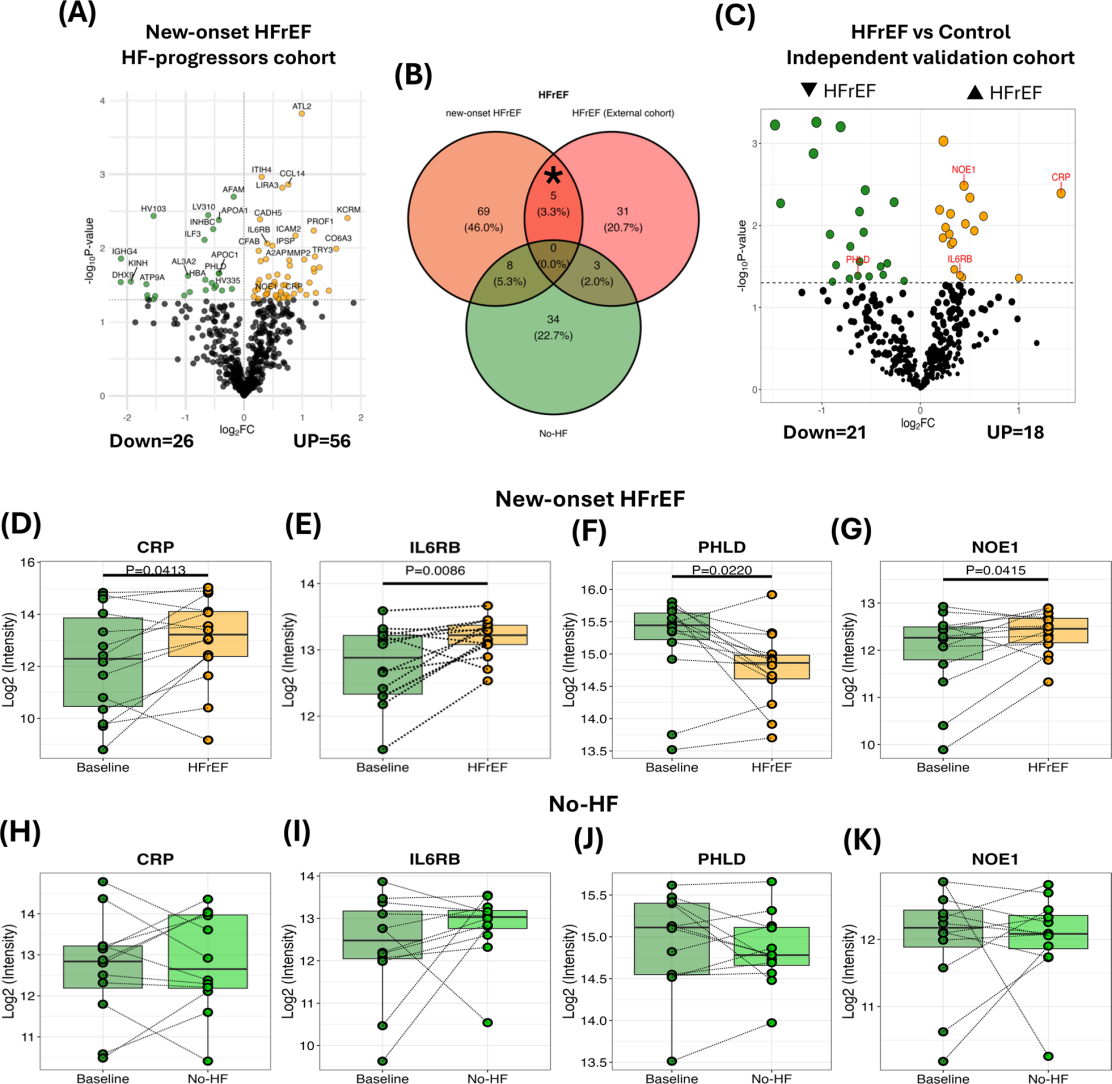
Biomarkers associated with HFrEF development. **A** Volcano plot of new-onset HFrEF, compared to baseline (yellow = up-regulated proteins; green = down-regulated proteins). **B** Venn diagram of differential protein expression (P < 0.05) for new-onset HFrEF, No-HF, and independent community HFrEF cohort. **C** Volcano plot of HFrEF vs control patients in independent community HFrEF cohort. **D**–**F** Co-expression proteins in new-onset HFrEF patients from (**B**) between new-onset HFrEF and independent community HFrEF cohort. **G**–**I** Co-expression proteins in No-HF patients from (**B**) between new-onset HFrEF and independent community HFrEF cohort. HFrEF = heart failure with reduced ejection fraction; No-HF = no heart failure; CRP = C-reactive protein; IL6RB = interleukin-6 receptor subunit beta; PHLD = phosphatidylinositol-glycan-specific phospholipase D; NOE1 = noelin. *Overlap protein expression in same direction between new-onset HFrEF and independent community HFrEF cohort

Of 82 significant differential proteins in new-onset HFrEF, 56 up-regulated proteins and 26 down-regulated proteins were identified at HFrEF diagnosis, compared to baseline (Fig. 4A). Five proteins, including C-reactive protein (CRP), interleukin-6 receptor subunit beta (IL6RB), phosphatidylinositol-glycan-specific phospholipase D (PHLD), noelin (NOE1), and apolipoprotein F (APOF) were overlapping between HFrEF progressors and the HFrEF patients in the independent community-based cohort (HFrEF vs control) (Fig. 4B, C). However, APOF increased in new-onset HFrEF in the STOP-HF cohort and decreased in HFrEF in the community-based cohort; this was therefore excluded from the candidate protein biomarker analysis for HFrEF development. Plasma PHLD level was significantly lower at HFrEF development than baseline (P = 0.0220; Fig. 4D), while CRP, IL6RB, and NOE1 were considerably higher in HFrEF progressors, compared to baseline (all P < 0.05; Fig. 4E–G). All of these signature proteins associated with HFrEF development did not change in No-HF group over time (all P > 0.05; Fig. 4H–K).

ORA was performed to gain insight into the biological processes of PHLD (encoded by GPLD1), CRP, IL6RB (encoded by IL6ST), and NOE1 (encoded by OLFM1) related to HFrEF development (Supplement Fig. 2). GPLD1 was enriched in various pathways involving biosynthetic and metabolic processes, localization, and transport (Supplement Fig. 2A). In Supplement Fig. 2B, OLFM1, IL6ST, and CRP were associated with positive regulation of gene expression. Furthermore, IL6ST and CRP were mainly involved in immune processes and responses (Supplement Fig. 2B).

**Fig. 5:**
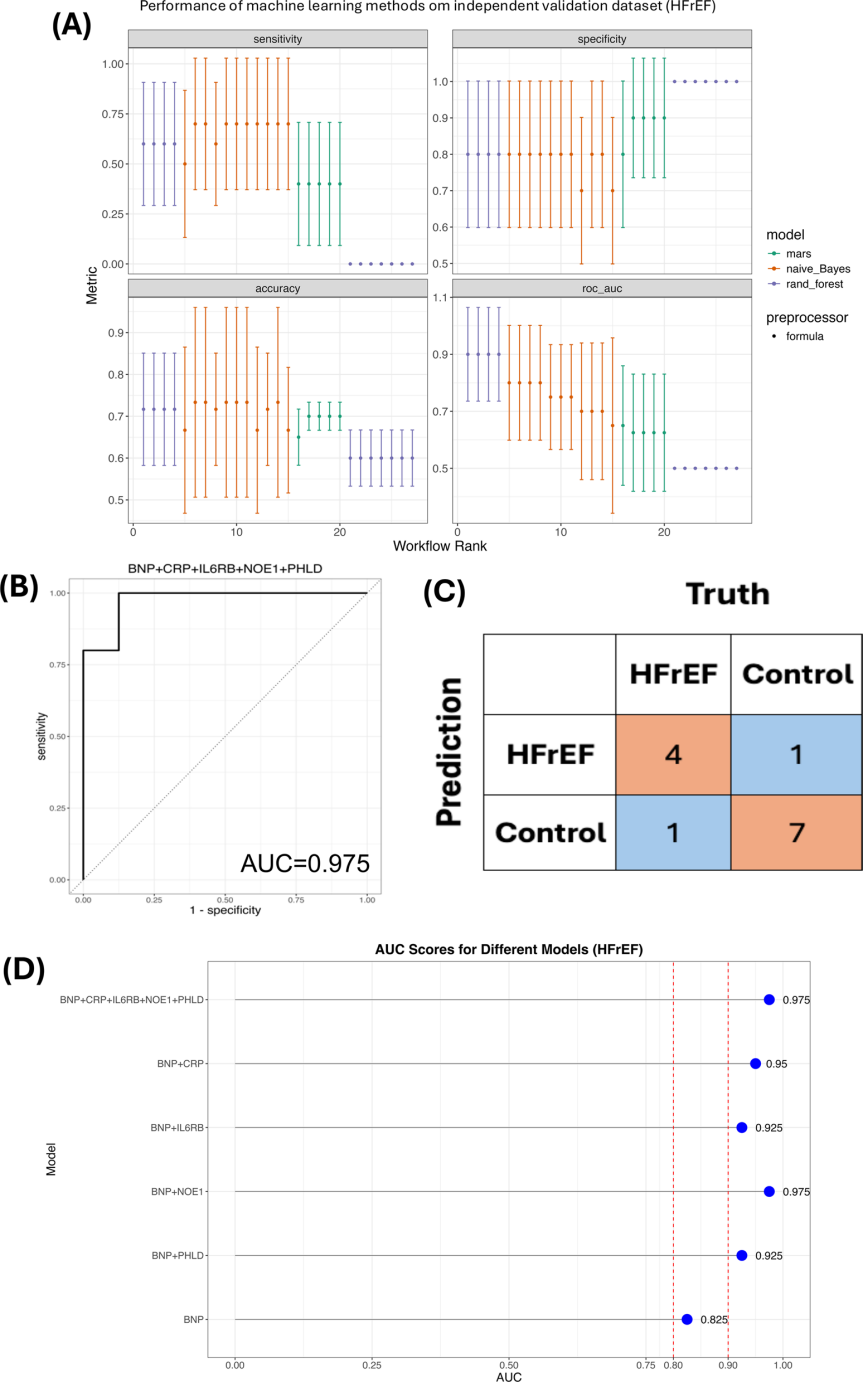
Predictive accuracy of plasma proteins related to HFrEF, alone or in combination with BNP measurement in independent community HFrEF cohort. **A** Models performance based on naive Bayes, multivariate adaptive regression splines (MARS), and random forest in training dataset. **B** Receiver operating curves show the performance of combining all candidate protein biomarkers with BNP measurement for predicting all incident HFrEF in testing dataset. **C** Confusion matrix of (**B**). **D** AUC of different combination or BNP alone in testing dataset. HFrEF = heart failure with reduced ejection fraction; CRP = C-reactive protein; IL6RB = interleukin-6 receptor subunit beta; PHLD = phosphatidylinositol-glycan-specific phospholipase D; NOE1 = noelin

We found that the RF algorithm demonstrated the best fit to the dataset based on the highest AUC in the training dataset, compared to MARS and NB (Fig. 5A). The panel of BNP + CRP + IL6RB + NOE1 + PHLD showed AUC at 0.975, and the confusion matrix exhibited one misclassification for HFrEF and healthy control (Fig. 5B, C). In general, combining our blood-based proteins associated with HFrEF development with BNP illustrated better performance for classifying HFrEF than using BNP alone: BNP (AUC = 0.825); BNP + NOE1 (AUC = 0.975); BNP + CRP (AUC = 0.950); BNP + IL6RB and BNP + PHLD (AUC = 0.925) (Fig. 5D).

## Discussion

By performing a mass-spectrometry-based discovery proteomics study, we identified plasma proteins associated with HFpEF and HFrEF development utilising serial blood samples from a new onset HF progressor cohort. We further evaluated differential plasma proteins associated with HF progression in an independent cohort of HF patients who attended a rapid access HF diagnostic clinic. It was found that IGF2, VCAM1, and ITIH3 were associated with HFpEF development, whilst PHLD, CRP, IL6RB, and NOE1 were associated with HFrEF. The biological processes of these proteins were revealed, and we subsequently applied machine learning to examine the discriminative ability of the plasma proteins related to the pathogenesis of HF subtypes when combined with BNP measurement, an established biomarker for HF diagnosis [9–12]. Generally, adding the novel plasma proteins to BNP significantly improved HF subtype prediction, which reflected the complementary information of panels of blood-based biomarkers. This underlines a plausible strategy using biomarker-based blood screening for early identification of HF subtypes, particularly in the setting of limited access to echocardiogram or magnetic resonance imaging.

Several studies emphasise the role of insulin, insulin like growth factor (IGF), and insulin like growth factor binding protein (IGFBP) pathway in HFpEF [29–32]. Consistent with previous research [32], this study showed that lower levels of IGF proteins, including IGF1 and IGF2, were linked to prevalent HFpEF and all-cause mortality. In a nested case–control study (n = 39,242), an increase of 1 standard deviation in IGF2 levels was associated with a 47% lower risk of mortality from HF [33]. The IGF axis, which is crucial for cellular proliferation and growth in various tissues, including the heart, can improve cardiac development, hypertrophy, and myocardial contractility [34–37]. Indeed, our analysis revealed that IGF2 is involved in several pathways that may influence HF outcomes, such as tissue development, cell differentiation and cellular processes. Our results showed an increase in VCAM1 and ITIH3 levels in HFpEF patients. Myocardial inflammation is central to the pathogenesis of HFpEF, and previous studies using endomyocardial biopsy samples from the LV of HFpEF patients have revealed elevated levels of inflammatory endothelial adhesion molecules, including VCAM1 in HFpEF [38, 39]. It was evident that reduced left atrial (LA) strain significantly linked VCAM-1 to incident HFpEF, suggesting elevated VCAM-1 impairs myocardial function, leading to HFpEF development [40]. Additionally, ITIH3 has been observed to be increased in HFpEF patients and concomitant atrial fibrillation, coronary microvascular disease and HFpEF [41]. Proteins associated with HF-risk factors were identified in the Atherosclerosis Risk in Communities (ARIC) study; ITIH3 was associated with incident HFpEF and was associated with larger LV (LVEDVI and LVESVI) and LA size (LAVI) [42]. Our machine learning analysis demonstrated that panels of BNP + IGF2, BNP + ITIH3, and BNP + VCAM1 + IGF2 + ITIH3 improved performance in discriminating HFpEF from other types of HF/no HF, compared to just using BNP alone. Similar AUCs were observed, suggesting that IGF2 and ITIH3 are the most informative biomarkers, while VCAM1 may not significantly enhance model performance. This observation highlights biomarker panels with optimised predictive value, emphasising the importance of selecting the most informative biomarkers for classification accuracy and clinical relevance.

On the other hand, 4 proteins were identified to play significantly important roles in HFrEF development in our dataset, including PHLD, CRP, IL6RB, and NOE1. In dilated cardiomyopathy (DCM), which is a common cause of HFrEF, PHLD (GPLD1) significantly decreased in DCM patients compared to controls [43], supporting our findings. Although plasma CRP, an inflammatory marker, was increased in HFrEF in the present study, CRP could also be a prognostic marker for HFpEF, HF with mildly reduced EF (HFmrEF), and HFrEF related to increased risks of in-hospital mortality [44]. In patients with established cardiovascular disease (CVD), elevated levels of CRP were independently linked to a higher long-term risk of developing HF [45]. Moreover, in the study of 4 longitudinal community-based cohorts [46], CRP was linked to incident HFrEF and was more markedly associated with HFrEF than HFpEF. Among inflammatory biomarkers in HF, biomarkers related to cytokine receptors such as IL6RB (IL6ST) were reportedly linked to HFrEF [47]. Elevated membrane glycoprotein 130 (gp130; one of the receptors for IL-6), an alternative name of IL6RB, is associated with worsening HF functional class, cardiovascular mortality, and death from HF [48, 49]. Taken together, these protein-related inflammatory processes are pivotal drivers in the pathogenesis of HFrEF, supporting the gradual increase in inflammation and remodelling. We found that NOE1 (OLFM1) was increased in HFrEF patients. There are few studies based on OLFM1 in HF development; however, it is known that OLFM1 is present in the embryonic heart and plays a role in heart development [50]. Furthermore, OLFM1, in cooperation with TGFβ, is involved in epithelial-mesenchymal transition (EMT), leading to an overproducing matrix to cause tissue stiffness in pathologic settings [51]. Another study has shown that using both an anti-OLFM1 antibody and siRNA that targeted OLFM1 inhibited the formation of mesenchymal cells [52]. Machine learning based on the RF algorithm in our analysis showed that adding these proteins to BNP added incremental discriminative capabilities to identify HFrEF patients beyond solely using BNP measurement. Interestingly, the combination of NOE1 and BNP improves classification performance for HFrEF in this dataset, as indicated by the AUC value, highlighting NOE1 as a significantly informative biomarker for HFrEF.

The strengths of our study include longitudinal proteomic analysis of HF progressors (from baseline to overt HF, either HFpEF or HFrEF) using untargeted mass spectrometry-based proteomics. We have also been able to verify the disease relevance of candidate biomarker proteins of interest in an independent cohort and evaluate the performance of biomarker panels using a machine learning approach. We acknowledge several limitations in this study, with the sample size being the most significant. The patients in this study were recruited from the Republic of Ireland, which may not be representative of the general global HF population. Thus, caution is warranted when generalising the findings to broader populations. While the dia-PASEF workflow offers a thorough evaluation of circulating proteins, it does not cover the entire human proteome, and there may be biases in prioritising the measurement of secreted proteins, and the number of identified proteins depends on proteins identified in libraries. Further studies are necessary to understand the mechanisms by which our protein biomarkers influence HF risk. Although blood-based biomarkers have emerged as an accessible, cost-effective, and highly promising tool for improving early HF detection, measuring specific biomarkers such as IL6RB and ITIH3 remains uncommon in clinical practice in some settings. Additionally, some of our identified biomarkers may contain several biological processes, such as ITIH3, which has also been previously associated with gastric cancer, hepatic steatosis, and psychiatric disorders [53–55]. Therefore, larger studies are needed to evaluate these biomarkers' sensitivity and specificity in accurately detecting HF subtypes using identified blood biomarkers in this study. Early identification of cardiac dysfunction is crucial to preventing the progression of HF. Patients with HFpEF and HFrEF exhibit distinct pathobiology [56]. Noninvasive methods, such as serological biomarkers, are valuable for estimating disease severity, predicting prognosis, and enabling the early detection of HF.

According to the American Heart Association/American College of Cardiology/Heart Failure Society of America (AHA/ACC/HFSA) and the European Society of Cardiology (ESC) guidelines, implementation of quadruple therapy with renin–angiotensin–aldosterone system (RAAS) inhibitors (including ARNIs or ACEIs or ARBs), β-blockers, MRAs and SGLT2 inhibitors is recommended as the first step in patients with HFrEF to ameliorate clinical progression, reduce HF hospitalisation, and improve survival as early as within 30 days of drug initiation [10, 12, 57]. Thus, the prompt initiation of quadruple therapy in HFrEF patients may be important in theory, similar to the importance of the timely initiation of induction chemotherapy in patients with cancer. There are currently limited effective therapies for patients with HFpEF. Recently, both guidelines are unified in their new recommendations for SGLT2 inhibitors in patients with HFpEF to reduce the risk of HF hospitalisation and cardiovascular death [10, 12, 57]. Distinguishing disease subtypes within HF patients is imperative to generate an understanding of unique HF subtypes and identify subgroups of patients who did not benefit from available treatment options. Altogether, our study emphasises the importance of blood-based biomarker development, which may enable early identification of HF subtypes and monitor treatment response. This enhances development of future HF subtype-specific medications.

Collectively, in the setting of limited resources for HF diagnosis, developing and combining our blood protein biomarkers with BNP measurement could improve diagnostic accuracy of HF and identify subtypes of HF, leading to early HF detection and initiation of GDMT, mitigating the poor prognosis of patients with HF.

## Conclusions

We analysed HF progressors who developed HFpEF or HFrEF and revealed a number of proteins and functional biological processes that potentially contributed to the pathogenesis of HFpEF and HFrEF, which were further verified in an independent cohort. The machine learning approach demonstrated that adding our plasma proteins to BNP principally improved HF subtype prediction.

## Supplementary Information

Additional file1 (DOCX 13921 KB)

## Data Availability

Data are available upon reasonable request from the corresponding author.
