# Supplementary material for: Proteomic-based biomarker discovery reveals panels of diagnostic biomarkers for early identification of heart failure subtypes
Source: J Transl Med. 2025 May 15;23:546. doi: 10.1186/s12967-025-06563-7 (PMC12082982; doi:10.1186/s12967-025-06563-7)
Supplement: Supplementary file 1 — Additional file1 (DOCX 13921 KB) [file 12967_2025_6563_MOESM1_ESM.docx]

Supplement for

**Proteomic-Based Biomarker Discovery Reveals Panels of Diagnostic Biomarkers for Early Identification of Heart Failure Subtypes**

Authors

Narainrit Karuna^1,2^, Claire Tonry^1^, Mark Ledwidge^3,4^, Nadezhda Glezeva^3^,
Joe Gallagher^3,4^, Ken McDonald^3,4^, Chris J Watson^1,3,4*^

^1^Wellcome-Wolfson Institute for Experimental Medicine, Queen’s University Belfast, Belfast, United Kingdom

^2^Faculty of Pharmacy, Chiang Mai University, Chiang Mai, Thailand

^3^UCD Conway Institute and Research and Innovation Programme for Chronic Disease, School of Medicine, University College Dublin, Ireland

^4^STOP-HF Unit, Department of Cardiology, St. Vincent's University Healthcare Group, Dublin, Ireland

**Corresponding author:** Chris J Watson, chris.watson@qub.ac.uk

Wellcome-Wolfson Institute for Experimental Medicine, Queen’s University Belfast, Belfast, United Kingdom

**Sample Preparation**

A total of 80 plasma samples, including HFrEF (Baseline=14; new-onset HFrEF=14), HFpEF (Baseline=14; new-onset HFpEF =14), and No-HF (Baseline=12; no-HF=12) from the STOP-HF trial^1^ and 52 plasma samples from independent validation cohort^2^ (HFrEF=12, HFpEF=22, and Control=18) were thawed on ice directly before the preparation for proteomic process. The plasma samples were prepared with a standard pipeline. Plasma samples were depleted of high abundant protein using High-Select™ Top14 Abundant Protein Depletion Resin (A36372, Thermo Scientific™) in a 96-well plate to improve the throughput of plasma processing at room temperature prior to digestion. 600 µl of resin was added to each well of a 96-well plate. After the resin settled to the bottom of the well, 30 µl of each plasma sample was added and placed the plate on a plate shaker with gentle shaking for 30 minutes at room temperature. Subsequently, the plates were coupled with a 96-well collection plate and centrifuged at 100 RCF for 5 minutes. Samples flow-through were collected and were concentrated with Genevac^TM^ miVac Centrifugal, Fisher Scientific, Sweden. Immunodepleted samples were diluted, and diluted samples were digested by 8 M urea/10 mM Tris-HCl (pH 8), subsequently quantified by a bicinchoninic acid assay (BCA, Pierce–Thermo Scientific™) for protein concentration. 50 µg of protein from each sample was resuspended to give ﬁnal concentrations of 50 mM ammonium bicarbonate (pH 7.8), and 5 µl of 100 mM dithiothreitol (DTT) was added into each well. Samples were incubated for 1 hour at 27°C in an incubator to allow protein denaturation. 5 µl of 140 mM iodoacetamide (IAA) was added and incubated in the dark at room temperature for 30 minutes. An additional 2 µl of DDT was added to quench the excess of IAA and diluted the reaction with 50 mM ammonium bicarbonate (pH 7.8) to reduce the urea concentration to below 2M for maximum digestion activity. Promega sequencing grade trypsin (V5111, Promega UK) was added to each sample with a protease to protein ratio of 1:50 and incubated overnight at 37 °C. The reaction was stopped by adding trifluoroacetic acid (TFA) to a final concentration of 1%, and samples were concentrated and stored at -20 °C. For peptide purification, stage tips were prepared in-house using commercially available C18 Empore Disks (3M, Minneapolis, MN). Samples were resuspended in 20 µl 1% TFA and checked concentration with on nanodrop nanodrop (Thermo Scientific). Stage tips were activated by the addition of 50 µl of 50% acetonitrile (AcN)/0.1% TFA and centrifugation at 1500 RCF for 10 min at room temperature. A 10 µl (~5 µg) of the sample was added into the stage tip and centrifuged at 1500 RCF at room temperature for 10 minutes. Then, stage tips were washed twice with 50 µl of 1% TFA centrifuged at 1500 RCF for 10 minutes each at room temperature. Peptides bound to the discs were eluted into clean low-bind Eppendorf tubes using a solution of 50% AcN/0.1%. Purified peptides were concentrated and resuspended with 25 µl of Solvent A (0.1% formic acid), stored at -20 °C until analyses.

**Mass spectrometry-based proteomics**

Samples (~1 µg) were injected into an Evosep One HPLC system (Evosep) coupled to a hybrid TIMS-quadrupole TOF mass spectrometer (Bruker Daltonik timsTOF Pro). Peptides were separated using a reversed-phase C18 PepSep column (15 cm x 100 µm ID, C18, 3 µm) with a preset 30 SPD method. The mobile phases were 0.1% (v/v) formic acid in water (phase A) and 0.1% (v/v) formic acid in acetonitrile (phase B). An increasing gradient of mobile phase B was applied over 44 minutes at a flow rate of 0.5 µl/min to achieve the separation.

In Data Dependent Acquisition (DDA) mode, the timsTOF Pro mass spectrometer operated in positive ion polarity with both TIMS (Trapped Ion Mobility Spectrometry) and PASEF (Parallel Accumulation Serial Fragmentation) modes enabled. The accumulation and ramp times for TIMS were set to 100 ms, and the ion mobility (1/k0) ranged from 0.6 to 1.6 Vs/cm. Spectra were recorded in the mass range of 100 to 1,700 m/z. The precursor (MS) intensity threshold was set at 2,500, and the precursor target intensity was set at 20,000. Each PASEF cycle consisted of one MS ramp for precursor detection followed by 10 PASEF MS/MS ramps, totalling a cycle time of 1.17 seconds.

In Data Independent Acquisition (DIA) mode on the timsTOF Pro, a diaPASEF scheme was created using the Bruker timsControl interface (version 2.0.53). This scheme consisted of 34 precursor isolation windows, each 26 Da wide with a 1 Da mass overlap, covering a mass range from 350 to 1,200 m/z and an ion mobility range from 0.6 to 1.6 Vs/cm.

**Proteomics analysis**

The spectral library built with FragPipe (v20.0), MSFragger (v3.8) and Philosopher (v5.0) from DDA PASEF runs and filtered at 1% protein and peptide FDR^3-5^. The DIA-NN (v1.8.1)^6^ was configured to operate with maximum mass accuracy tolerances of 15 ppm for both MS1 and MS2 spectra. For analyses utilising DDA-based spectral libraries, protein inference was disabled to use the protein groups within those libraries. MBR was enabled, and Quantification mode was set to “Robust LC (high precision)”. All other settings were left default. The software output was filtered with a precursor q-value of less than 1%. Additionally, a global protein q-value filter of less than 1% was applied.

The data tables processed from DIA-NN were utilised for analysis, focusing on the Protein.Group column in DIA-NN's report to identify the protein groups. To stabilize variance and normalize the distribution, the data underwent a log2 transformation. To ensure reliability, proteins that were observed in less than 50% of the samples were removed from the dataset. Normalisation was then performed using the LOESS method from the limma package^7^ using normalizeBetweenArrays() to correct for any systematic biases present in the data.

For handling missing values, any remaining missing values were imputed using random draws from a manually defined left-shifted Gaussian distribution, with a shift parameter of 1.8 and a scale parameter of 0.3, to simulate realistic values. The analysis proceeded with the identification of differentially expressed proteins using either a t-test or a paired t-test, depending on the experimental design and data structure. The p-values were adjusted using the Benjamini and Hochberg (BH) method.

**Statistical analysis for proteomics**

Paired t-test was used to compare proteins changes at diagnosis of HF subtypes from baseline within HF progressors (STOP-HF trial), whilst comparison of 2 groups used independent t-test (independent validation cohort).

**Pathway analysis**

Over-representation analysis (ORA) was conducted to reveal the biological processes associated with significantly differentially expressed proteins in subtypes of HF from baseline, with a significance threshold of P values less than 0.05. The analysis utilised the clusterProfiler package^8^, with specific parameters set as follows: the minimum gene set size (minGSSize) was set to 2, the maximum gene set size (maxGSSize) was set to 500, and the p-value cutoff (pvalueCutoff) was set to 0.05. The ontology (ont) parameter was set to "BP" for biological processes. The outcomes of this analysis were visualized using the SRplot platform^9^, allowing for comprehensive representation of the biological processes linked to the differentially expressed proteins in the HF subtypes.

**Machine learning approach for biomarker models**

Machine learning analyses were carried out using the tidymodels package, version 1.2, available at <https://CRAN.R-project.org/package=tidymodels>. This package was chosen for its comprehensive suite of tools for building, tuning, and evaluating machine learning models.

To minimise the risk of overfitting, accurately estimate prediction performance, and fine-tune model hyperparameters, we utilised a fivefold cross-validation approach. This method involves dividing the dataset into five equal parts, training the model on four parts, and validating it on the remaining part. This process is repeated five times, with each part used as the validation set once. The results are then averaged to provide a more reliable estimate of the model's performance.

Three different machine learning algorithms were employed to find the best classifier for the dataset: naive Bayes (NB)^10^, multivariate adaptive regression splines (MARS)^11^, and random forest (RF)^12^. Naive Bayes is a simple probabilistic classifier based on Bayes' theorem, which is particularly effective for large datasets. MARS is a non-parametric regression technique that can model complex relationships by fitting piecewise linear regressions. Random forest is an ensemble learning method that constructs multiple decision trees and merges their results to improve predictive accuracy and control overfitting.

An independent validation cohort served as the input data and BNP measurement together with candidate blood-based biomarkers for either HFpEF or HFrEF were variables (predictors). The dataset was split into two parts: 60% for training and 40% for testing. The training data was used to estimate model parameters, with and without hyperparameter tuning, which involves adjusting the model's settings to optimise performance. The models' performance during training was assessed using the area under the receiver operating characteristic curve (AUC) during cross-validation. The model with the highest AUC was considered the best-trained model.

Finally, the best-trained models were tested on the testing dataset. The Receiver Operating Characteristic (ROC) curve, along with the AUC, was used to evaluate the diagnostic performance and accuracy of the prediction models. The ROC curve is a graphical representation of a model's ability to discriminate between positive and negative classes, and the AUC provides a single metric to summarize this performance. This evaluation process ensured that the selected models were both accurate and reliable for making predictions on new data.

**Reference**

1. Ledwidge, M.*, et al.* Natriuretic peptide-based screening and collaborative care for heart failure: the STOP-HF randomized trial. *JAMA* **310**, 66-74 (2013).

2. James, S.*, et al.* Life expectancy for community-based patients with heart failure from time of diagnosis. *Int J Cardiol* **178**, 268-274 (2015).

3. da Veiga Leprevost, F.*, et al.* Philosopher: a versatile toolkit for shotgun proteomics data analysis. *Nat Methods* **17**, 869-870 (2020).

4. Kong, A.T., Leprevost, F.V., Avtonomov, D.M., Mellacheruvu, D. & Nesvizhskii, A.I. MSFragger: ultrafast and comprehensive peptide identification in mass spectrometry-based proteomics. *Nat Methods* **14**, 513-520 (2017).

5. Yu, F.*, et al.* Analysis of DIA proteomics data using MSFragger-DIA and FragPipe computational platform. *Nat Commun* **14**, 4154 (2023).

6. Demichev, V., Messner, C.B., Vernardis, S.I., Lilley, K.S. & Ralser, M. DIA-NN: neural networks and interference correction enable deep proteome coverage in high throughput. *Nat Methods* **17**, 41-44 (2020).

7. Ritchie, M.E.*, et al.* limma powers differential expression analyses for RNA-sequencing and microarray studies. *Nucleic Acids Res* **43**, e47 (2015).

8. Wu, T.*, et al.* clusterProfiler 4.0: A universal enrichment tool for interpreting omics data. *Innovation (Camb)* **2**, 100141 (2021).

9. Tang, D.*, et al.* SRplot: A free online platform for data visualization and graphing. *PLoS One* **18**, e0294236 (2023).

10. Kuhn, M. & Johnson, K. *Applied predictive modeling*, (Springer, 2013).

11. Friedman, J.H. & Roosen, C.B. An introduction to multivariate adaptive regression splines. *Stat Methods Med Res* **4**, 197-217 (1995).

12. Wright, M.N. & Ziegler, A. ranger: A fast implementation of random forests for high dimensional data in C++ and R. *arXiv preprint arXiv:1508.04409* (2015).

**Supplement Figure 1**


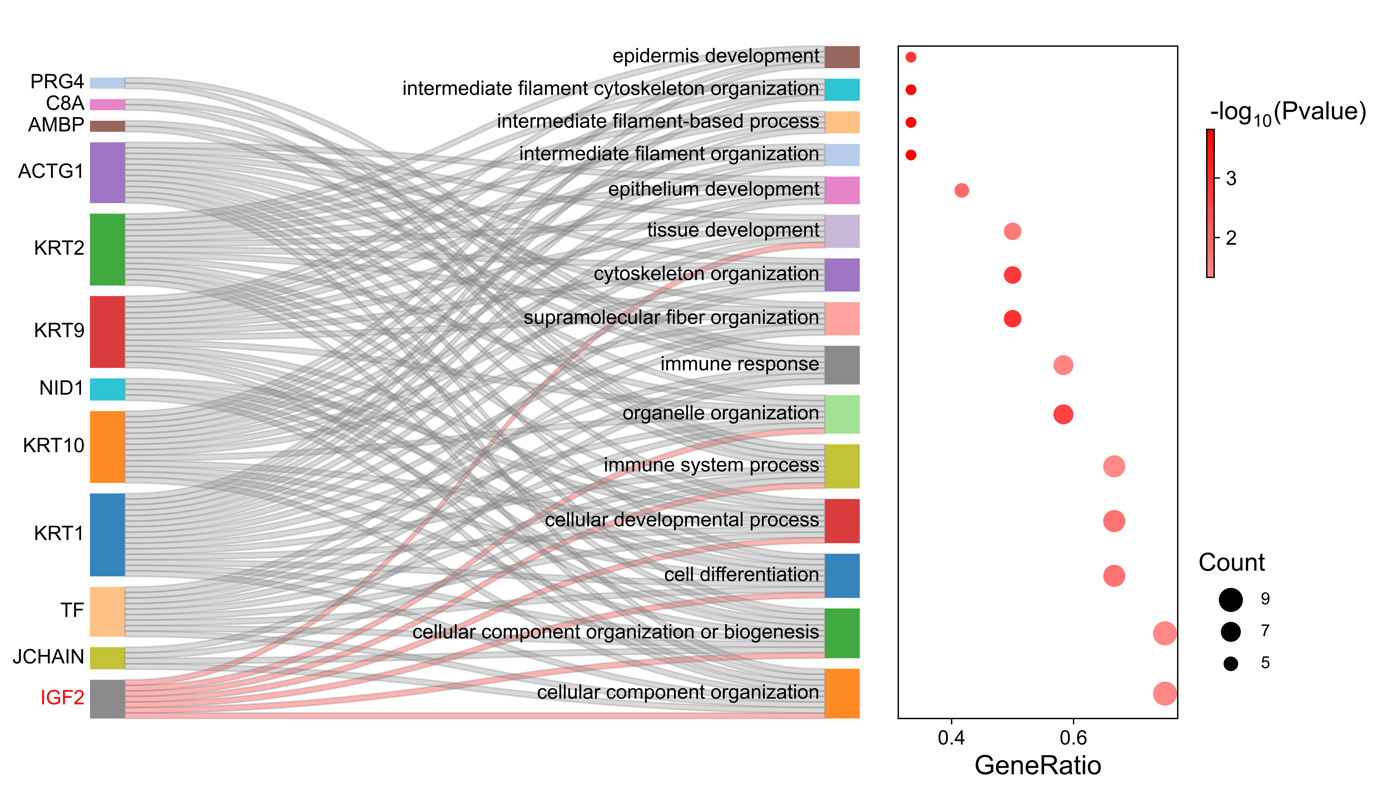


**(A)**

**(B)**


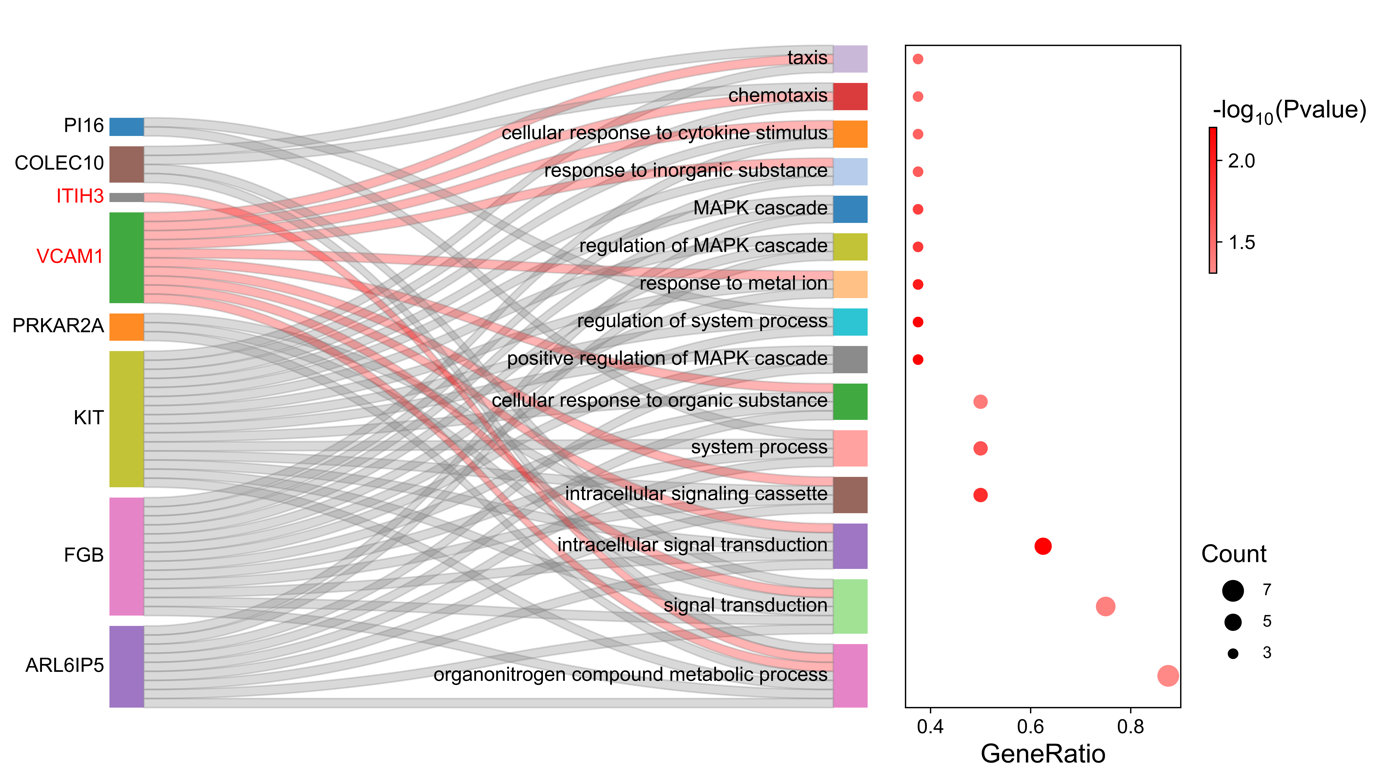


**Supplement Figure 1: Gene ontology analysis of biological processes of candidate biomarkers for HFpEF.** (A) Over representative analysis of down-regulated proteins in HFpEF. (B) Over representative analysis of up-regulated proteins in HFpEF. Red=candidate biomarkers associated with HFpEF development. HFpEF=heart failure with preserved ejection fraction; IGF2= insulin like growth factor 2; VCAM1= vascular cell adhesion protein 1; ITIH3=inter-alpha-trypsin inhibitor heavy chain 3.

**Supplement Figure 2**

**(A)**


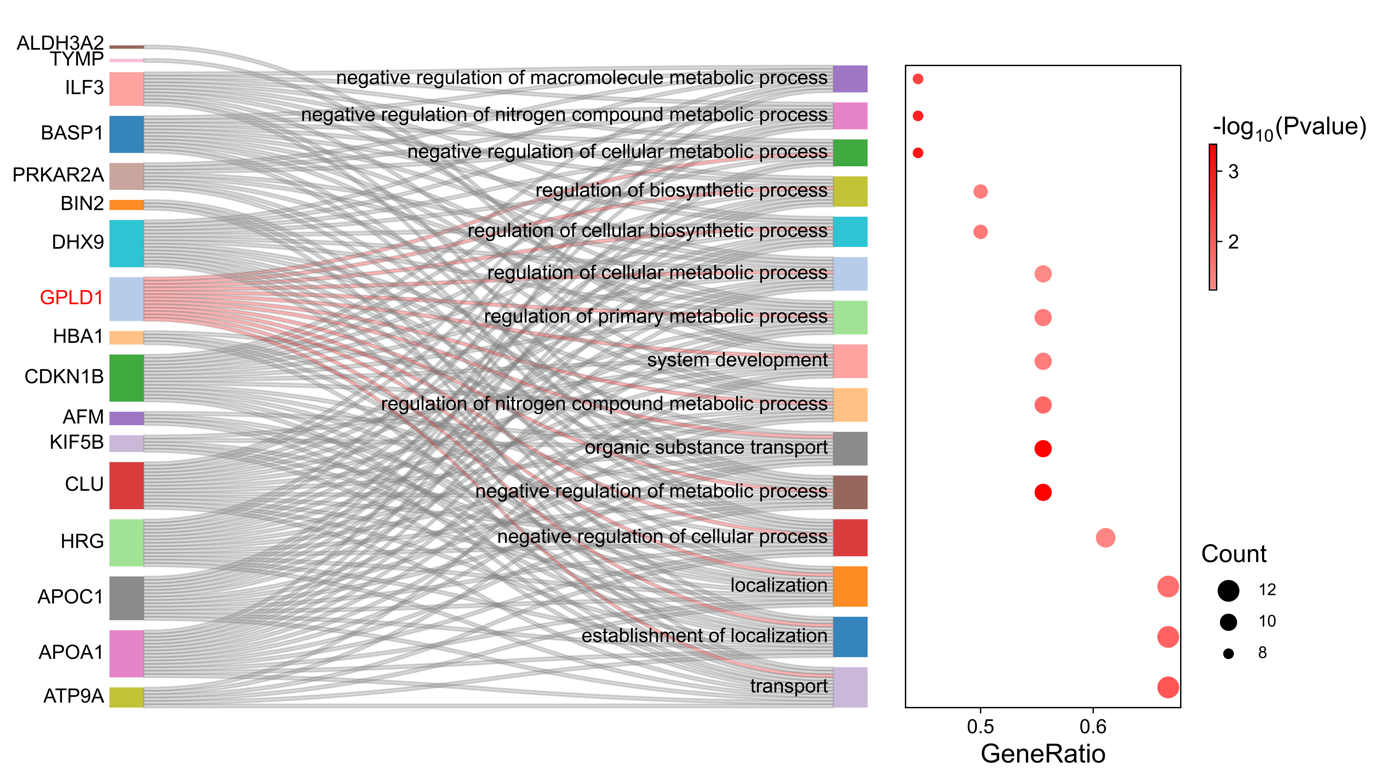


**(B)**


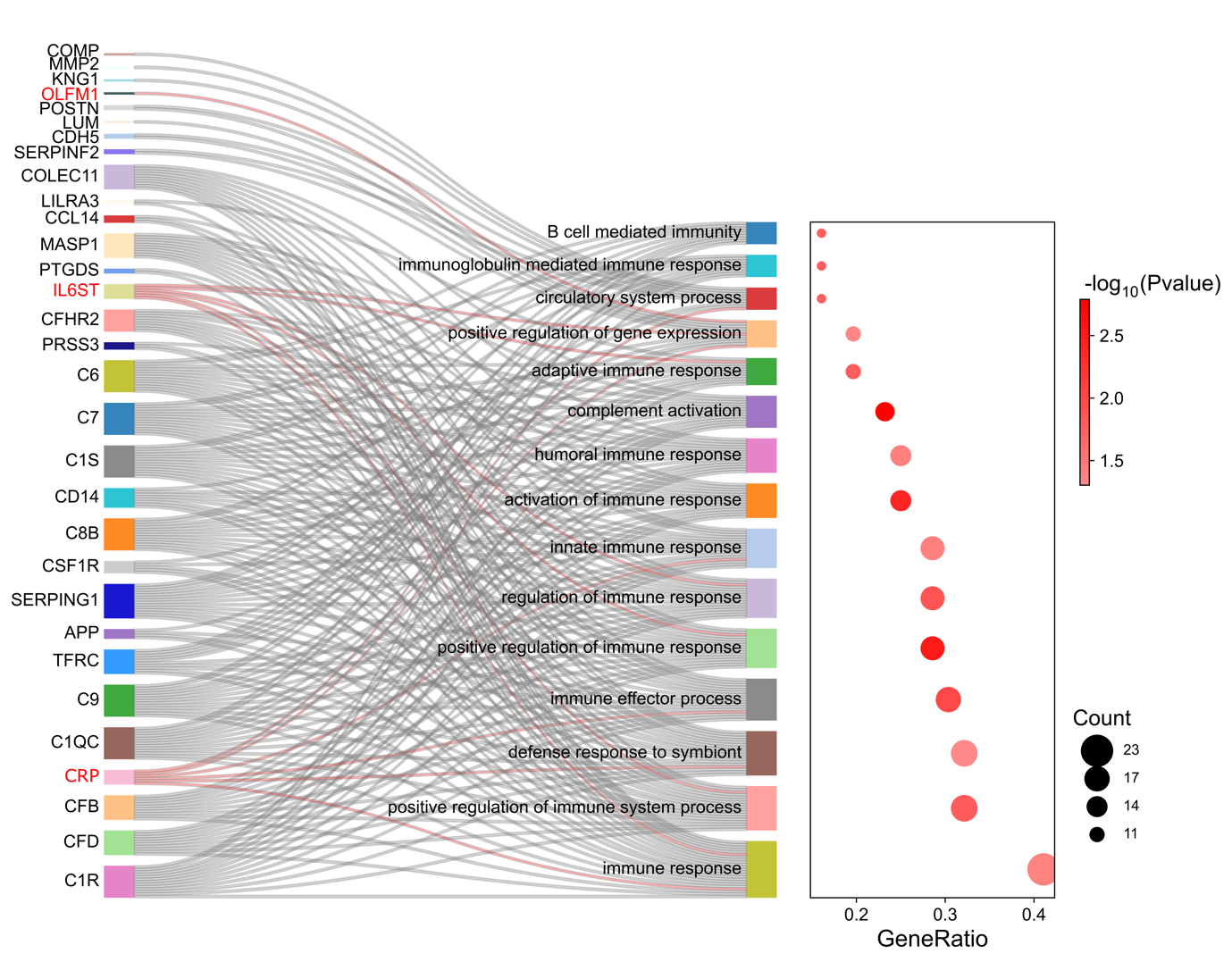


**Supplement Figure 2: Gene ontology analysis of biological processes of candidate biomarkers for HFrEF.** (A) Over representative analysis of down-regulated proteins in HFrEF. (B) Over representative analysis of up-regulated proteins in HFrEF. Red=candidate biomarkers associated with HFrEF development. HFrEF=heart failure with reduced ejection fraction; CRP=C-reactive protein; IL6RB (encoded by IL6ST)=interleukin-6 receptor subunit beta; PHLD (encoded by GLPD1)=phosphatidylinositol-glycan-specific phospholipase D; NOE1 (OLFM1)=noelin.
